# The Combined Effect of Perceived COVID-19 Infection Risk at Work and Identification with Work Community on Psychosocial Wellbeing among Finnish Social Sector and Health Care Workers

**DOI:** 10.3390/ijerph17207623

**Published:** 2020-10-19

**Authors:** Eerika Finell, Annukka Vainio

**Affiliations:** 1 Faculty of Social Sciences, Tampere University, 33100 Tampere, Finland; 2 Helsinki Institute of Sustainability Science, Faculty of Agriculture and Forestry, University of Helsinki, 00014 Helsinki, Finland; annukka.vainio@helsinki.fi

**Keywords:** COVID-19, coronavirus pandemic, shared identity, risk perception, work identification, stress, psychosocial well-being, health care workers, social workers

## Abstract

It has been well documented that both risk perception and group identification are related to psychosocial well-being. However, their combined effect has rarely been analyzed. We examined the combined effect of perceived risk associated with COVID-19 infection at work and work community identification on psychosocial well-being (i.e., frequency of stress symptoms) among health care and social sector workers in Finland (*N* = 1279). Data were collected via an online questionnaire in June 2020 and analyses of covariance were conducted. Perceived COVID-19 infection risk at work was classified into high, medium and low risk. In total, 41% of participants reported a high risk. After all background variables were included, participants who reported high perceived infection risk and low work community identification reported stress symptoms more often than those who reported high perceived risk and high identification (*p* = 0.010). Similarly, the former differed significantly from all other comparison groups (medium and low risk, *p* < 0.001), being the most stressed. We found that perceived infection risk and work community identification were not related to each other. Our conclusion is that high work community identification can buffer employee stress when faced with a high perceived health risk. In the context of the COVID-19 pandemic, work organizations with a high infection risk should advance the possibility of employees’ identification with their work community.

## 1. Introduction

A new coronavirus disease, caused by the SARS-CoV-2 virus and identified as COVID-19, was first acknowledged in China in 2019 and rapidly became a global threat to the health of many employees working in the social and health care sectors. There is evidence that COVID-19 has a disproportional impact on health care workers both physically and psychologically, causing a high incidence of disease, death, and a number of psychological problems, including stress, depression and anxiety [1,2,3,4]. Although psychological symptoms have been found to be particularly high among those working on the frontline during the pandemic [5,6,7,8], non-frontline heath care workers have also reported such symptoms [9]. Moreover, these symptoms have been more severe among females and younger workers [4,10,11], as well as for those with prior mental health problems [12,13]. In addition to these cases, social sector workers, who have seldom been acknowledged in the recent COVID-19 literature, have also faced risk of infection in their work [14]. For example, in England and Wales, social care workers had a significantly raised rate of death between March and May 2020 [2].

It is clear that COVID-19 is causing psychological stress to social sector and health care workers due to the increased risk of personnel infection, fear of spreading the disease, concern for one’s own family if one becomes sick [3], lack of knowledge on the disease as well as deaths among other professionals [15]. On the one hand, perceived health risk is important in order to motivate individuals to engage in health-protective behaviors [16]. On the other hand, the perceived risk is associated with increased stress symptoms, in particular when the management of the risk is at least partly beyond one’s own behavioral control [17,18]. Among health care and social sector workers, the perceived infection risk is dependent on the protective measures taken by organizational infrastructures and governments. Accordingly, confidence in protective measures has been found to be associated with reduced psychological symptoms [13,19,20].

Various protective strategies against COVID-19-related occupational stress and its negative effects have been considered, ranging from resilience enhancement to providing adequate organizational support systems [12,13,14]. Curiously, in the context of social sector and health care workers, the role of organizational group processes has rarely been discussed. According to the “social cure” perspective to social identity, group membership has a positive impact on well-being and health [21]—an association shown to be prevalent among medical service workers [22]. Groups can provide powerful psychological resources to their members, such as social support, which can reduce stress symptoms [23,24,25]. For example, people who identify with a group perceive other group members’ social support more positively than people who do not. In addition, those reporting high levels of work community identification are more confident of receiving support from other group members when needed than those less inclined to identify [23,25]. Thus, when occupational organizations consider protective strategies against stress that is related to COVID-19 infection risk at work among social sector and health care workers, promoting identification with their work community might be one key protective factor that can buffer this stress.

This article analyses the combined effect of the perceived risk associated with COVID-19 infection at work with work community identification on psychosocial well-being (i.e., frequency of stress symptoms) among health care and social sector workers in Finland. We analyze both groups because in Finland, social sector and health care workers often work in the same organizations and institutions as part of multi-professional teams. To our knowledge, the buffering effect of group identification on the relationship between perceived health risk and stress symptoms has yet to be empirically tested. Drawing on the “social cure” perspective [25], we test our main hypothesis that those social sector and health care workers who report both a high perceived COVID-19 infection risk at work and low work community identification suffer significantly more often from stress symptoms than those who report high perceived COVID-19 infection risk and high work community identification.

## 2. Materials and Methods

### 2.1. Participants and Procedure

The data were collected through The Union of Health and Social Care Professionals in Finland, which distributed the invitation and the link to an online questionnaire to its members. In total, 1555 members filled in and submitted the online questionnaire, with a response rate of 5%. In the first page, participants were informed about the aim of the research and had the opportunity to scroll through the form before answering questions; participants were not obliged to respond to any of the items, ensuring full voluntarism could be guaranteed, and informed consent was positively required. Only participants who provided informed consent, were in paid or salaried employment, and were 18–64 years old were included. The final sample included 1279 participants. The data were collected between 28 May and 16 June 2020 using the Limesurvey program. The Ethics Committee of the Tampere Region approved this study (41/2020).

### 2.2. Materials

#### 2.2.1. Outcome Variable

The outcome measure was self-reported stress symptoms (i.e., stress symptoms) measured by four items: “during last two months, how frequently you have suffered from”: (a) fatigue, apathy or lack of energy; (b) difficulties in falling asleep or recurrent awakenings at night; (c) tenseness, nervousness or irritability; (d) feeling that it is “all just too much”? These items were drawn from the Finnish Quality of Work Life Survey [26] and have often been used as indicators of stress and mental exhaustion [27,28]. In these analyses, the items were measured on a five-point scale, with the number selected corresponding to the frequency of symptoms experienced: 0 = never, 1 = less often, 2 = once or twice a month, 3 = a few times a week, and 4 = almost daily. The items were summed and then divided by the total number of items the participants responded to. The rate was calculated if at least two items were answered. The reliability was good (Cronbach’s alpha = 0.84).

#### 2.2.2. Predictors

The perceived risk associated with COVID-19 at work (i.e., perceived risk at work) was measured by one item: How likely do you consider the following things will happen in relation to your work in six months: I will have COVID-19 infection in my workplace. A similar item has been used elsewhere [29]. See also [30]. The response scale varied from “very likely” (1) to “very unlikely” (5). Responses reporting that the risk had already materialized (N = 6), as well as those who reported that the question did not apply to them (N = 82), were coded as missing values. The item was recategorized with 0 corresponding to the risk is unlikely (values 4 and 5 in the original scale), 1 corresponding to the risk is intermediate (value 3 in the original scale) and 2 corresponding to the risk is likely (values 1 and 2 in the original scale).

The work community identification was measured by two items adapted by Doosje et al. (“I feel strong ties with other members of my work community”, “My work community is an important part of me”) [31,32]. The five response options varied between ‘totally disagree’ (1) and ‘totally agree’ (7). The items were summed and divided by the number of items (mean = 5.11, standard deviation (SD) = 1.43). The rate was calculated if at least one item was answered. Responses reporting that the question did not apply to them (N = 18) were coded as missing values. The reliability was good (Cronbach alpha = 0.88). Then, the summed variable was recategorized into two categories, using the mean as a cut-off value (0 = low identification; 1 = high identification).

Finally, we formed one new combined variable. We combined perceived risk at work and work community identification as follows: 1 = low risk and high identification, 2 = low risk and low identification, 3 = medium risk and high identification, 4 = medium risk and low identification, 5 = high risk and high identification, and 6 = high risk and low identification.

#### 2.2.3. Background Variables

The background variables were gender, age, number of people living in the household, highest level of education, occupational group, working time pattern (e.g., two-shift work, three-shift work) and whether the participant belonged to a COVID-19 infection risk group. The working time pattern was controlled because shift work is associated with insomnia [33]. The occupational group was determined through the following response options: 0 = social sector work and 1 = health care work. If requested, participants had the possibility to report another occupational group. In total, 11 participants reported that they worked both in social sector and health care work. Due to the small frequency of this latter category, it was not included in the analysis and was coded as a missing value [34]. A further 9 participants reported that they worked in the education sector. These participants were categorized alongside those who worked in the social sector (0 = social sector work, 1 = health care work). In addition, we controlled the perceived risk associated with COVID-19 in one’s spare time (i.e., perceived risk in spare time): How likely do you consider the following thing will happen in your spare time in next six months: I will have COVID-19 infection in my spare time. The response scale varied from very likely (1) to very unlikely (5). The item was recategorized as the perceived risk at work, as explained above. Responses reporting that the risk had already materialized (*N* = 6) were coded as missing values. Finally, we assessed trust in the Finnish authorities by a single item: I trust the Finnish authorities in their treatment of the coronavirus pandemic. The response scale varied from totally agree (1) to totally disagree (5).

### 2.3. Statistical Analyses

The effects of all the variables on perceived risk at work and work community identification were examined using cross-tabulations and analyses of variance. The effects of all the variables on stress symptoms were examined using analyses of variance and Pearson correlation coefficients. The main and combined effects of perceived risk at work and work community identification on stress symptoms were subjected to an analysis of covariance (ANCOVA). This analysis was carried out using IBM SPSS Statistics for Windows, Version 25.0. (IBM Corp., Armonk, NY, USA) The missing data were handled by using listwise deletion. The percentage of missing data varied from 0% (age) to 10% (perceived risk in spare time). The mean of the missing values was 2% per variable.

## 3. Results

The majority of the participants were women, aged between 35 and 44 years, and had tertiary education. Table 1 presents the frequencies and means of all the variables, as well as their associations with the predictors and the outcome variable.

In total, 41% of participants reported a high risk of COVID-19 infection at work. Perceived risk at work was significantly associated with all background variables with the exception of gender, highest education, occupational group, and belonging to a COVID-19 risk group (see Table 1). The risk perception decreased with age, so that whilst 67% of the youngest age group reported a high risk, only 32% of those in the age group 55–64 reported the same. Those who reported medium risk lived in significantly bigger households (mean = 3.20, SD = 1.36) than those who reported low (mean = 2.92, SD = 1.30) or high risk (mean = 3.01, SD = 1.36). A higher percentage of those who had two-shift work (48%), three-shift work (46%) or who worked atypical hours (49%) reported a higher COVID-19 infection at work than those who had regular day (36%) or evening/night work (23%). In addition, 18% of participants who reported high perceived risk at work also reported high perceived risk in their spare time, whereas the percentage among medium- and low-risk groups was 8% and 7%, respectively. Finally, those who reported high risk had significantly less trust in the Finnish authorities to effectively manage the Coronavirus pandemic (mean = 2.44, SD = 1.05) than those who reported low (mean = 2.18, SD = 1.07) or medium risk (mean = 2.28, SD = 0.90). There was no significant association found between perceived risk and work identification.

From the background variables, the work community identification was only significantly associated with working time patterns and having trust in the Finnish authorities (see Table 1). Those who had three-shift work, or worked atypical hours, reported higher work identification (56% and 68%, respectively) than those in two-shift work (50%), regular day work (48%), or regular evening or night work (21%). Finally, those with low work identification had less trust in the Finnish authorities to manage the pandemic (mean = 2.45, SD = 1.04) than those with high identification (mean = 2.22, SD = 0.96).

Stress symptoms were significantly associated with all the background variables except the number of people living in the household, occupational group, working time pattern and perceived risk in spare time (see Table 1). Women reported stress symptoms (mean = 2.29, SD = 0.93) more often than men (mean = 2.01, SD = 0.96), participants from the age group 25–34 reported symptoms the most often (mean = 2.52, SD = 0.87) and their stress level differed significantly from all the others except those in the 18–24 age group. Similarly, those who had secondary education reported these symptoms more often (mean = 2.42, SD = 0.93) than those with tertiary education (mean = 2.24, SD = 0.94). In addition, participants who belonged to a COVID-19 risk group reported stress symptoms more often (mean = 2.42, SD = 0.97) than those who did not (mean = 2.22, SD = 0.93). Finally, the lower the level of trust participants reported in the Finnish authorities to manage the Coronavirus pandemic, the greater the propensity to report stress symptoms.

Both predictors were significantly associated with stress symptoms (see Table 1). Participants who perceived a high risk of COVID-19 infection in their workplace reported stress symptoms more often than those who perceived only a medium or low risk. This association was still significant after all the background variables were included in the model (F(2, 1001) = 30.84, *p* < 0.001, ηp^2^ = 0.06). Estimated marginal means and standard errors of unadjusted and adjusted models are reported in Table 2 below.

Similarly, participants with low work identification reported stress symptoms more often than those with high work identification. Once again, this association was still significant after all the background variables were included in the model (F(1, 1018) = 13.87, *p* < 0.001, ηp^2^ = 0.01). Estimated marginal means and standard errors of unadjusted and adjusted models are reported in Table 3.

In light of these results, we examined the combined effect of perceived risk at work and work community identification on stress symptoms. The estimated marginal means, standard errors and pairwise comparisons of unadjusted and adjusted models are reported in Table 4. In the unadjusted model, the combined effect was significantly associated with stress symptoms (F(5, 1178) = 22.01, *p* < 0.001, ηp ^2^ = 0.09). Participants who reported high perceived risk and low work identification reported stress symptoms with significantly greater frequency than those who reported high perceived risk and high identification. All the mean differences between this category and other combined categories were significant. After inserting all the background variables into the model, the combined effect was still significantly associated with stress symptoms (F(5, 997) = 15.25, *p* < 0.001, ηp^2^ = 0.07). Similarly, the means of participants who reported both high risk and low work identification still differed significantly from all the other categories. There were no statistically significant interactions between perceived risk and work identification in the unadjusted (F(2, 1178) = 0.43, *p* = 0.650) or fully adjusted models (F(2, 986) = 0.49, *p* = 0.611).

## 4. Discussion

Our results support the hypothesis that those with low work community identification and high perceived COVID-19 infection risk at work show greater frequencies of stress symptoms than those with high identification and high perceived infection risk. The former group differed significantly from all other comparison groups in reporting the most stress symptoms. In addition, participants who reported high work community identification and low perceived COVID-19 infection risk at work reported significantly fewer stress symptoms than any other group. Finally, participants who reported a medium infection risk and low identification only showed significant differences from those who reported either high risk and low identification or no stressors at all (e.g., no risk and high identification). These findings show that higher levels of work community identification may act as a buffer against stress factors in the field of social sector and health care work, especially when employees perceive a high health risk in their workplace. As the social cure model argues, group identification is an important source of security, support and belonging and its effect on well-being has been demonstrated in many studies [25]. To our knowledge, however, this is the first study which has demonstrated this relationship in the context of the COVID-19 pandemic and risk perception.

Our results contribute to the extant literature in a number of other ways. First, the findings lend support to previous analyses reporting a link between risk perceptions and stress [17,18] by showing a strong relationship between higher perceptions of COVID-19 infection risk in the workplace and the increased frequency of stress symptoms. Secondly, work community identification and stress symptoms were also related. Participants reporting low levels of work community identification also reported stress symptoms more often than those reporting high levels of work community identification. This also supports findings that have been found before, albeit in a different context [35]. In contrast, work community identification and COVID-19 infection risk at work were not significantly associated with each other. This contradicts previous research suggesting that the greater the identification with a group, the lower the perceived risk is likely to be [36,37]. This claim was predicated on the basis that group identification increases trust. Although it is likely that this occurs in particular contexts (e.g., family celebrations) [38], this is unlikely to be the case in all circumstances. One important factor that might explain this inconsistency is the degree to which people are able to control their risk. In our cases, social sector and health care workers rarely have sufficient control over the risk factors, or necessary resources, that are able to make meaningful reductions to their perceived risk (e.g., proximity to infection; having enough personal protective equipment). Thus, people can report high levels of work community identification, and exhibit high levels of trust, but if the loci of risk controls are out of reach of the community, then group identification is unlikely to relate positively to perceptions of risk. This opens an important avenue for future research, namely, to explore in greater detail, and in more diverse contexts, how levels of control over risk-management resources affect the relationships between group identification and risk perception.

Finally, empirical research that has analyzed the well-being of social sector workers during COVID-19 pandemic is rare [14]. Our social sector and health care workers did not significantly differ in risk perception, work community identification or frequencies of stress symptoms. These occupational groups often work in the same organizations and institutions as part of multi-professional teams and thus share the same COVID-19 risk. More research analyzing the well-being of social sector workers is needed in the context of COVID-19.

Our results have strong practical implications. The findings can make a valuable contribution to the development of strategies for social sector and health care professionals to maintain their well-being under stressful working conditions during the COVID-19 pandemic. Given that risk perception and stress are mutually related [17,18], their interplay may produce a vicious circle which can end up in exhaustion and burn out. The social cure approach suggests that developing greater group identity is a powerful psychological resource in a such context [25]. Our results highlight the role of work community identification in contributing to social sector and health care workers’ ability to cope with stressors that cannot be avoided at work. For example, employers can strengthen the sense of community in their organizations by providing opportunities to share experiences within the group in a safe way and develop mutual trust as well as by recognizing and acknowledging the powerful role of collective strategies that the work community has successfully used in the past and adapting them to current environment [22].

Naturally, our findings have their limitations. The data are cross-sectional; thus, our reasoning is strongly based on previous research and theories. In addition, although our sample was representative of members of The Union of Health and Social Care Professionals in Finland, the response rate was lower than we would have preferred, and it is possible that those with higher levels of concern about their work condition had a greater motivation to answer than those who perceived that their work conditions were risk free or of low risk. These factors limit the generalizability of the results. In addition, the effect size was quite small. Although work community identification may act as a buffer, practical factors such as having enough personal protective equipment are of course essential for controlling the stress caused by COVID-19 infection risk. Cross-cultural, longitudinal and experimental data are needed to confirm the findings.

## 5. Conclusions

Our findings have shown that participants who report low work community identification and high perceived COVID-19 infection risk at work were significantly more stressed than other participants. This finding indicates that the combined effect of work community identification and risk perception needs to be considered in efforts to reduce the psychological stress social sector and health care workers face during the COVID-19 pandemic. Our findings provide further support to the notion that group processes are essential to people’s psychosocial well-being and that this is especially salient in times of health crises.

## Figures and Tables

**Table 1:** Descriptive statistics of all variables and their unadjusted associations with work identification, perceived COVID-19 risk at work, and stress symptoms (*N* = 1159–1279).

	*N*	% or Mean (SD)	Perceived Risk X^2^/F-Test	Work Identification X^2^/F-Test	Stress Symptoms F-Test/ Correlation
**Predictors**					
Work identification					
Low	621	49	3.40	-	22.72 ***^,g^
High	653	51			
Perceived risk at work					
Low	278	23	-	3.40	44.92 ***^,g^
Medium	421	35			
High	490	41			
**Outcome Variable**					
Stress symptoms ^a^	1272	2.27 (0.94) ^c,d^	44.92 ***^,g^	22.72 ***^,g^	-
**Background Variables**					
Gender					
Female	1168	93	1.38	0.02	7.25 **^,g^
Male	88	7			
Age (years)					
18–24	15	1			
25–34	227	18	35.69 ***	0.42	7.55 ***^,g^
35–44	443	35			
45–54	361	28			
55–64	233	18			
Number of people living in the household	1262	3.03 (1.35) ^c,e^	4.38 *^,g^	0.09 ^g^	0.00 ^h^
Level of highest education					
Secondary	246	19	5.22	1.72	7.27 **^,g^
Tertiary	1023	81			
Occupational group					
Social sector workers	174	14	0.59	3.17	0.02 ^g^
Health care workers	1091	86			
Working time pattern					
Regular day work	612	48	16.79 *	16.85 **	0.72 ^g^
Regular evening/night work	14	1			
Two-shift work	230	18			
Three-shift work	373	29			
Other working time pattern	47	4			
Belonging to a COVID-19 risk group			3.10	0.00	8.91 **^,g^
Yes	257	21			
No	953	79			
Perceived risk in spare time					
Low	592	51	93.65 ***	0.24	2.74 ^g^
Medium	428	37			
High	139	12			
Trust in the Finnish authorities ^b^	1252	2.33 (1.01) ^c,f^	6.53 **^,g^	15.34 ***^,g^	0.10 **^,h^

^a^ Scale 0–4; a higher number indicates symptoms reported more often; ^b^ scale 1–5, a higher number indicates lower trust; ^c^ mean (standard deviation); ^d^ Min. = 0, Max. = 4; ^e^ Min. = 1, Max. = 9; ^f^ Min. = 1, Max. = 5; ^g^ F-test; ^h^ Pearson correlation. *** <0.001, ** <0.01, and * <0.05.

**Table 2:** Unadjusted and adjusted effects of perceived COVID-19 risk on stress symptoms (*N* = 1016–1187).

Perceived COVID-19 Risk Unadjusted Model	*N*	Estimated Marginal Mean	Standard Error	Pairwise Comparison
1. Low	278	1.89	0.05	1 < 2 ***, 1 < 3 ***
2. Medium	419	2.23	0.04	2 > 1 ***, 2 < 3 ***
3. High	490	2.53	0.04	3 > 2 ***, 3 > 1 ***
**Perceived COVID-19 Risk** **Adjusted Model (Demographics) ^1^**	***N***	**Estimated Marginal Mean**	**Standard Error**	**Pairwise Comparison**
1. Low	268	1.92	0.06	1 < 2 ***, 1 < 3 ***
2. Medium	405	2.24	0.04	2 > 1 ***, 2 < 3 ***
3. High	471	2.52	0.04	3 > 2 ***, 3 > 1 ***
**Perceived COVID-19 Risk** **Adjusted Model (Demographics and Work Characteristics) ^2^**	***N***	**Estimated Marginal Mean**	**Standard Error**	**Pairwise Comparison**
1. Low	265	1.93	0.08	1 < 2 ***, 1 < 3 ***
2. Medium	399	2.24	0.07	2 > 1 ***, 2 < 3 ***
3. High	466	2.53	0.07	3 > 2 ***, 3 > 1 ***
**Perceived COVID-19 Risk** **Adjusted Model (Demographics, Work Characteristics, Member of Risk Group, Trust in Authorities and Perceived Risk in Spare Time) ^3^**	***N***	**Estimated Marginal Mean**	**Standard Error**	**Pairwise Comparison**
1. Low	239	1.94	0.08	1 < 2 ***, 1 < 3 ***
2. Medium	351	2.21	0.08	2 > 1 ***, 2 < 3 ***
3. High	426	2.52	0.08	3 > 2 ***, 3 > 1 ***

*** *p* < 0.001; ^1^ adjusted model controlled for gender, age, number of people living in the household, highest education; ^2^ adjusted model controlled for gender, age, number of people living in the household, highest education, occupational group, working time pattern; ^3^ fully adjusted model controlled for gender, age, number of people living in the household, highest education, occupational group, working time pattern, belonging to a COVID-19 risk group, perceived risk in spare time, and trust in the Finnish authorities.

**Table 3:** Unadjusted and adjusted effects of work community identification on stress symptoms (*N* = 1032–1267).

Work Community Identification Unadjusted Model	*N*	Estimated Marginal Mean	Standard Error	Pairwise Comparison (*p*-Value)
1. Low	618	2.40	0.04	1 > 2 ***
2. High	649	2.15	0.04	
**Work Community Identification** **Adjusted Model (Demographics) ^1^**	***N***	**Estimated Marginal Mean**	**Standard Error**	**Pairwise Comparison** **(*p*-Value)**
1. Low	600	2.39	0.04	1 > 2 ***
2. High	621	2.16	0.04	
**Work Community Identification** **Adjusted Model (Demographics and Work Characteristics) ^2^**	***N***	**Estimated Marginal Mean**	**Standard Error**	**Pairwise Comparison** **(*p*-Value)**
1. Low	589	2.39	0.07	1 > 2 ***
2. High	617	2.15	0.07	
**Work Community Identification** **Adjusted Model (Demographics, Work Characteristics, Member of Risk Group, Trust in Authorities and Perceived Risk in Spare Time) ^3^**	***N***	**Estimated Marginal Mean**	**Standard Error**	**Pairwise Comparison** **(*p*-Value)**
1. Low	500	2.37	0.07	1 > 2 ***
2. High	532	2.16	0.07	

*** *p* < 0.001; ^1^ adjusted model controlled for gender, age, number of people living in the household, highest education; ^2^ adjusted model controlled for gender, age, number of people living in the household, highest education, occupational group, working time pattern; ^3^ fully adjusted model controlled for gender, age, number of people living in the household, highest education, occupational group, working time pattern, belonging to a COVID-19 risk group, perceived risk in spare time, and trust in the Finnish authorities.

**Table 4:** Unadjusted and adjusted combined effects of perceived COVID-19 infection risk and work community identification on stress symptoms (*N* = 1015–1184).

Unadjusted Model	*N*	Estimated Marginal Means	Standard Error	Pairwise Comparisons
1. Low risk + high identification	156	1.75	0.07	1 < 2 **^,a^, 1 < 3 ***, 1 < 4 ***, 1 < 5 ***, 1 < 6 ***
2. Low risk + low identification	121	2.07	0.08	2 > 1 **^,a^, 2 < 4 *^,b^, 2 < 5 ***, 2 < 6 ***
3. Medium risk + high identification	206	2.13	0.06	3 > 1 ***, 3 < 4 *^,c^, 3 < 5 ***, 3 < 6 ***
4. Medium risk + low identification	211	2.33	0.06	4 > 1 ***, 4 > 2 *^,b^, 4 > 3 *^,c^, 4 < 6 ***
5. High risk + high identification	249	2.43	0.06	5 > 1 ***, 5 > 2 ***, 5 > 3 ***, 5 < 6 *^,d^
6. High risk + low identification	241	2.63	0.06	6 > 1 ***, 6 > 2 ***, 6 > 3 ***, 6 < 4 ***, 6 < 5 *^,d^
**Adjusted Model (Demographics) ^1^**	***N***	**Estimated Marginal Means**	**Standard Error**	**Pairwise Comparisons**
1. Low risk + high identification	150	1.80	0.07	1 < 2 **^,e^, 1 < 3 ***, 1 < 4 ***, 1 < 5 ***, 1 < 6 ***
2. Low risk + low identification	117	2.08	0.08	2 > 1 **^,e^, 2 < 4 *^,f^, 2 < 5 **^,g^, 2 < 6 ***
3. Medium risk + high identification	199	2.14	0.06	3 > 1 ***, 3 < 4 *^,h^, 3 < 5 **^,i^, 3 < 6 ***
4. Medium risk + low identification	204	2.32	0.06	4 > 1 ***, 4 > 2 *^,f^, 4 > 3 *^,h^, 4 < 6 **^,g^
5. High risk + high identification	236	2.41	0.06	5 > 1 ***, 5 > 2 **^,g^, 5 > 3 **^,i^, 5 < 6 *^,j^
6. High risk + low identification	235	2.62	0.06	6 > 1 ***, 6 > 2 ***, 6 > 3 ***, 6 < 4 **^,g^, 6 < 5 *^,j^
**Adjusted Model (Demographics and Work Characteristics) ^2^**	***N***	**Estimated Marginal Means**	**Standard Error**	**Pairwise Comparisons**
1. Low risk + high identification	148	1.79	0.09	1 < 2 **^,k^, 1 < 3 ***, 1 < 4 ***, 1 < 5 ***, 1 < 6 ***
2. Low risk + low identification	116	2.09	0.10	2 > 1 **^,k^, 2 < 4 *^,l^, 2 < 5 **^,g^, 2 < 6 ***
3. Medium risk + high identification	198	2.15	0.08	3 > 1 ***, 3 < 5 **^,i^, 3 < 6 ***
4. Medium risk + low identification	199	2.32	0.08	4 > 1 ***, 4 > 2 *^,l^, 4 < 6 ***
5. High risk + high identification	236	2.41	0.08	5 > 1 ***, 5 > 2 **^,g^, 5 > 3 **^,i^, 5 < 6 *^,b^
6. High risk + low identification	230	2.63	0.08	6 > 1 ***, 6 > 2 ***, 6 > 3 ***, 6 < 4 ***, 6 < 5 *^,b^
**Adjusted Model (Demographics, Work Characteristics, Member of Risk Group, Trust in Authorities and Perceived Risk in Spare Time) ^3^**	***N***	**Estimated Marginal Means**	**Standard Error**	**Pairwise Comparisons**
1. Low risk + high identification	133	1.82	0.10	1 < 2 *^,m^, 1 < 3 **^,i^, 1 < 4 ***, 1 < 5 ***, 1 < 6 ***
2. Low risk + low identification	106	2.09	0.11	2 > 1 *^,m^, 2 < 5 **^,n^, 2 < 6 ***
3. Medium risk + high identification	172	2.14	0.09	3 > 1 **^,i^, 3 < 5 **^,a^, 3 < 6 ***
4. Medium risk + low identification	178	2.27	0.09	4 > 1 ***, 4 < 6 ***
5. High risk + high identification	219	2.40	0.09	5 > 1 ***, 5 > 2 **^,n^, 5 > 3 **^a^, 5 < 6 *^,b^
6. High risk + low identification	207	2.63	0.09	6 > 1 ***, 6 > 2 ***, 6 > 3 ***, 6 < 4 ***, 6 < 5 *^,b^

*** <0.001, ** <0.01, and * <0.05. ^a^ *p* = 0.004, ^b^ *p* = 0.010, ^c^ *p* = 0.022, ^d^ *p* = 0.015, ^e^ *p* = 0.009, ^f^ *p* = 0.018, ^g^ *p* = 0.001, ^h^ *p* = 0.041, ^i^ *p* = 0.002, ^j^ *p* = 0.014, ^k^ *p* = 0.007, ^l^ *p* = 0.026, ^m^ *p* = 0.021, and ^n^ *p* = 0.003; ^1^ adjusted model controlled for gender, age, number of people living in the household, highest education; ^2^ adjusted model controlled for gender, age, number of people living in the household, highest education, occupational group, working time pattern; ^3^ fully adjusted model controlled for gender, age, number of people living in the household, highest education, occupational group, working time pattern, belonging to a COVID-19 risk group, perceived risk in spare time, and trust in the Finnish authorities.
